# The external validity of published randomized controlled trials in primary care

**DOI:** 10.1186/1471-2296-10-5

**Published:** 2009-01-19

**Authors:** Ritu Jones, Robert O Jones, Colin McCowan, Alan A Montgomery, Tom Fahey

**Affiliations:** 1 Division of Community Health Sciences, University of Dundee, MacKenzie Building, Kirsty Semple Way, Dundee, DD2 4BF, UK; 2 Department of Community Based Medicine, University of Bristol, 25 Belgrave Road, Bristol, BS8 2AA, UK; 3 Department of General Practice and Family Medicine, Division of Population Health Sciences, Royal College of Surgeons in Ireland, Beaux Lane House, Mercer Street Lower, Dublin 2, Ireland

## Abstract

**Background:**

A criticism of Randomized Controlled Trials (RCTs) in primary care is that they lack external validity, participants being unrepresentative of the wider population. Our aim was to determine whether published primary care-based RCTs report information about how the study sample is assembled, and whether this is associated with RCT characteristics.

**Methods:**

We reviewed RCTs published in four primary care journals in the years 2001–2004. Main outcomes were: (1) eligibility fraction (proportion eligible of those screened), (2) enrolment fraction (proportion randomised of those eligible), (3) recruitment fraction (proportion of potential participants actually randomised), and (4) number of patients needed to be screened (NNS) in order to randomize one participant.

**Results:**

A total of 148 RCTs were reviewed. One hundred and three trials (70%) reported the number of individuals assessed by investigators for eligibility, 119 (80%) reported the number eligible for participation, and all reported the actual number recruited. The median eligibility fraction was 83% (IQR 40% to 100%), and the median enrolment fraction was 74% (IQR 49% to 92%). The median NNS was 2.43, with some trials reportedly recruiting every patient or practice screened for eligibility, and one trial screening 484 for each patient recruited. We found no association between NNS and journal, trial size, multi- or single-centre, funding source or type of intervention. There may be associations between provision of sufficient recruitment data for the calculation of NNS and funding source and type of intervention.

**Conclusion:**

RCTs reporting recruitment data in primary care suggest that once screened for eligibility and found to match inclusion criteria patients are likely to be randomized. This finding needs to be treated with caution as it may represent inadequate identification or reporting of the eligible population. A substantial minority of RCTs did not provide sufficient information about the patient recruitment process.

## Background

Randomized controlled trials (RCTs) are regarded as the gold standard for determining the effectiveness of medical interventions.[1] In order to improve the quality of RCT's, reporting standards have been developed that relate to the design, conduct, analysis and interpretation when submitting a report on an RCT to a peer-review journal. These explicit standards- the Consolidated Standards of Reporting Trials (CONSORT) enables readers to understand an RCT's conduct and assess the validity of its results.[2]

The issue of external validity or generalizability of an RCT arises when judgements have to be made in relation to applying RCT evidence to an individual patient.[3,4] The more similar a patient is to those recruited to an RCT, the more confident a clinician can be in applying that RCT's results back to an individual patient. Unfortunately, there is accumulating evidence that participants recruited to RCTs differ in important aspects to those patients seen in primary care settings. For example, patients in RCTs of hypertension are more often younger, male, at lower cardiovascular risk and have less co-morbidity than patients in primary care.[5,6] In the case of patients with asthma or chronic obstructive pulmonary disease over 90% would not have met inclusion criteria for RCTs on which treatment recommendations and clinical guidelines are based.[7,8]

The CONSORT checklist focuses primarily on improved reporting of information relating to the internal validity of an RCT.[2] The revised template for the CONSORT flow diagram starts at the stage of asking investigators to state the number of participants who were assessed for eligibility for the RCT. This assumes that all eligible individuals were approached to participate in an RCT. Most researchers know that this is usually not the case, particularly when investigators rely on clinicians to recruit incident cases in situations when clinical contact time is short- for example in patients with acute respiratory illness.[9] For non-acute illness, ensuring high external validity can be equally challenging; research ethics committees now insist that approaches to patients to take part in research requires that initial contact be made through family practices, not through the research team. This limits the ability of researchers to know whether or not the complete target population have been approached to take part in their research study. RCTs based in primary care have shown that there is a highly variable rate of recruitment within individual family physicians and between individual family practices.[10,11]

The aim of this study was to assess the external validity of RCTs published in four primary care journals by quantifying the selection process of trial enrolees from the primary care population.

## Methods

### Data sources and collection

We reviewed RCTs published in four primary care journals in the years 2000–2004: *BMJ *(primary care section), *British Journal of General Practice*, *Family Practice *and *The Journal of Family Practice*.

We collected the following data fields from each RCT:

• Citation details- title, journal citation; author contact email.

• Characteristics of RCT and funding source- individual, cluster based, factorial, cross-over RCT; number practices/patients recruited per arm of RCT; number and type of interventions; country and setting of RCT; funding source- public (NIH, NHS, MRC, Wellcome Trust, Charity), pharmaceutical, self funding.

• Features concerning patient population and recruitment process- inclusion/exclusion criteria in RCT; statement concerning target population; potential population; eligible population and participation; details concerning target population engagement; details concerning eligibility screening; details concerning enrolment of eligible population; statement whether incident cases or prevalent cases were recruited; how patient population was identified- during consultation, disease/practice register, other method; statement of how and who approached eligible cases – by family physician, researcher, other; statement of method of where and in what circumstances eligible population were enrolled- family physician during clinic, family physician by post, practice nurse, waiting room, other; control group event rate.

### Data extraction

Two data extractors (RJ and ROJ) independently assessed and extracted relevant data from each published RCT. A Microsoft ACCESS^® ^database was used to collate relevant details and the completed dataset was imported into Stata^® ^statistical package for analysis. Disagreement was resolved involvement of a third author (TF) and a consensus decision reached. CM and AM analysed the dataset, independent of the two data extractors. Table 1 provides definitions of the RCT recruitment terminology. Eligibility, enrolment and recruitment fractions were calculated in the same way as Gross et al.[12]

**Table 1:** RCT Recruitment Terminology

**Term**	**Definition**
Target population	Location and characteristics of potentially eligible individuals, representing the patient population to whom the results of the RCT are expected to apply
Eligibility fraction	Proportion of potential participants who undergo screening and are eligible to enrol in the RCT
Enrolment fraction	Proportion of people who are eligible for participation and who actually enrol in the RCT
Recruitment fraction	Proportion of potential participants who actually enrol in the RCT
Number of patients needed to be screened (NNS)	Number of patients screened in order to randomise one participant (equal to 1/recruitment fraction)

### Statistical Analysis

We summarized RCT characteristics as proportions and compared groups using the chi-squared test. We calculated the median Number Needed to Screen (NNS) to randomize one participant and assessed if the NNS, and reporting of data required to calculate the NNS, was related to the following explanatory variables: journal in which the RCT was published; number of enrollees in the RCT; whether the RCT was single or multicentre; funding source and the type of intervention studied.

## Results

### Descriptive statistics

A total of 148 RCTs were reviewed. As three of the four journals assessed are published in the UK, it was not surprising to find that the majority of RCTs originated from there (56%), with other significant contributions from the US (10%) and the Netherlands (8%). The main funding source was government agency (57%), most were multi-centre (79%), and 17% had at least 650 participants. One hundred and three RCTs (70%) reported the number of individuals assessed by investigators for eligibility, 119 (80%) reported the number eligible for participation, and all reported the actual number recruited (Table 2).

**Table 2:** Characteristics of included RCTs in terms of total, eligible and recruited population *

**Characteristic**	**Total Trials (%)**	**Trials That Reported Target Population**	**Trials That Reported Number of Patients Eligible**
**Total RCTs**	148 (100)	103 (70)	119 (80)
**Journal**			
BMJ	58 (39)	44 (39)	50 (42)
British Journal of General Practice	46 (31.0)	31 (30)	36 (30)
Family Practice	24 (16)	17 (15)	19 (16)
Journal of Family Practice	20 (14)	11 (17)	14 (12)
**Number of enrolees**			
<100	48 (32)	40 (39)	44 (37)
110–260	51 (34)	31 (30)	37 (31)
260–650	24 (16)	15 (15)	19 (16)
>650	25 (17)	17 (17)	19 (16)
**Multicentre**			
Multiple	116 (79)	83 (81)	94 (80)
Single	27 (18)	16 (16)	21 (18)
Unclear	4 (3)	3 (3)	3 (3)
**Funding Source**			
Charity	15 (13)	10 (12)	13 (14)
Governing body	5 (4)	3 (4)	3 (3)
Government	66 (57)	55 (65)	60 (63)
Pharmaceutical Industry	15 (12)	8 (9)	10 (10)
Other	14 (13)	9 (11)	10 (10)
Intervention Studied			
Counselling/Lifestyle	11 (8)	8 (8)	10 (8)
Decision Support aids	7 (5)	6 (6)	7 (6)
Educational	26 (18)	21 (21)	23 (14)
Pharmaceutical therapy	23 (16)	9 (9)	11 (9)
Surgery/procedure	3 (2.0)	3 (3)	3 (3)
Other	76 (52)	55 (54)	64 (54)

### Recruitment statistics

Of the trials that reported quantitative recruitment information, the median eligibility fraction was 83% (IQR 40% to 100%), and the median enrolment fraction was 74% (IQR 49% to 92%). The recruitment fraction could be calculated for 103 trials (70%), with a median participant recruitment of 41% (IQR 16% to 71%) (Table 3). The median NNS was 2.43 (IQR 1.41 to 6.25), with some trials reportedly recruiting every patient or practice screened for eligibility, and one trial screening 484 for each patient recruited. We found no association between magnitude of the NNS and journal publication, RCT size, multi- or single-centre RCT, funding source or type of intervention (Table 4). The data suggested that trials funded by government of interventions other than pharmaceutical therapies may be more likely to provide sufficient recruitment data for calculation of NNS (Table 5).

**Table 3:** Eligibility, enrolment and recruitment fractions (n = 155)

**Data**	**Trials That Reported Data**	**Median**	**Interquartile Range**
	n		%
**Eligibility Fraction**	102	83	40–100
**Enrolment Fraction**	119	74	49–92
**Recruitment Fraction**	103	41	16–71

**Table 4:** Relationship between Number of patients Needed to Screen (NNS) to identify one participant and RCT characteristics

**Characteristics**	**Total Trials**	**Median Number Needed to Screen to Identify One Participant (Interquartile Range)**	**P Value**
Total	103		
Journal			0.78
BMJ	44	2.95 (1.42–9.30)	
British Journal of General Practice	31	2.30 (1.38–4.42)	
Family Practice	17	1.81 (1.23–3.46)	
Journal of Family Practice	11	2.79 (1.95–7.92)	
Number of enrolees			0.40
<100	40	3.15 (1.63–5.64)	
110–260	31	2.19 (1.22–9.95)	
260–650	15	1.66 (1.32–3.11)	
>650	17	2.78 (1.38–5.16)	
Multicentre			
Multiple	83	2.79 (1.42–6.33)	
Single	16	2.05 (1.18–7.83)	
Unclear	3	1.66 (1.60–5.95)	
**Funding Source**			0.09
Charity	10	2.61 (1.23–4.48)	
Governing body	3	1.60(1.60–6.06)	
Government	55	2.14 (1.34–6.33)	
Pharma Industry	8	3.98 (2.10–15.59)	
Other	9	5.33 (1.95–7.92)	
Not Listed	18	2.62 (1.23–3.48)	
**Intervention Studied**			0.47
Counselling/Lifestyle	8	4.75 (2.05–169.71)	
Decision Support aids	6	2.89 (1.42–17.21)	
Educational	21	2.05 (1.34–3.63)	
Pharmaceutical therapy	9	1.89 (1.66–5.16)	
Surgery/procedure	3	1.59 (1.11–9.95)	
Other	55	2.79 (1.42–6.38)	

**Table 5:** Relationship between provision of data required to calculate NNS and RCT characteristics

**Characteristics**	**Data required to calculate NNS provided in report, n(%)**		**Odds Ratio (95% CI)**	**P Value**
	NO	YES		
	(n = 45)	(n = 103)		
**Journal**				0.37
BMJ	14 (31)	44 (43)	-	
British Journal of General Practice	15 (33)	31 (30)	0.66 (0.28 to 1.56)	
Family Practice	7 (16)	17 (16)	0.77 (0.27 to 2.24)	
Journal of Family Practice	9 (20)	11 (11)	0.39 (0.13 to 1.13)	
**Number of enrolees**				0.09
<100	8 (18)	40 (39)	-	
110–260	20 (44)	31 (30)	0.31 (0.12 to 0.80)	
260–650	9 (20)	15 (15)	0.33 (0.11 to 1.02)	
>650	8 (18)	17 (16)	0.42 (0.14 to 1.32)	
**Multicentre**				0.45
Multiple	33 (73)	83 (81)	-	
Single	11 (24)	16 (16)	0.58 (0.24 to 1.38)	
Unclear	1 (2)	3 (3)	1.19 (0.12 to 11.88)	
Funding Source				
Charity	5 (11)	10 (10)	-	
Governing body	2 (4)	3 (3)	0.75 (0.09 to 6.04)	
Government	11 (24)	55 (53)	2.5 (0.71 to 8.76)	
Pharmaceutical Industry	7 (16)	8 (8)	0.57 (0.13 to 2.50)	
Other	5 (11)	9 (9)	0.9 (0.19 to 4.17)	
Not Listed	15 (33)	18 (17)	0.6 (0.17 to 2.14)	
**Intervention Studied**				0.02
Counselling/Lifestyle	3 (7)	8 (8)	-	
Decision Support aids	1 (2)	6 (6)	2.25 (0.18 to 27.37)	
Educational	5(11)	21 (21)	1.56 (0.30 to 8.17)	
Pharmaceutical therapy	14 (32)	9 (9)	0.24 (0.05 to 1.16)	
Surgery/procedure	0 (0)	3 (3)	undefined	
Other	21 (48)	55 (54)	0.98 (0.24 to 4.06)	

## Discussion

### Findings

We found that the reporting of recruitment to RCTs based in primary care is inconsistent and frequently incomplete. Of the 148 RCTs published, 103 (70%) reported the number of individuals who were screened for eligibility and 80% of published RCTs reported the number of individuals who were eligible. In those RCTs that did report on the recruitment process there appears to be marked variation in terms of the proportion of individuals who are recruited. These findings suggest that reporting of RCTs should be improved and that for some RCTs external validity is limited because only a low proportion of eligible participants are successfully recruited.

### Context of previous studies

Our findings differ with those of a recent study that report on the recruitment process in 172 RCTs published in four general medical journals- *Annals of Internal Medicine*, *JAMA*, *The New England Journal of Medicine *and *The Lancet*.[12] In this study half of the published RCTS were industry sponsored and over two thirds were of a pharmaceutical intervention, 52% of RCTs reported the number of persons who were evaluated by investigators for eligibility compared with 70% of primary-care based RCTs; similarly 43% of the RCTs published in general medical journals reported on the number of persons who were actually eligible for participation, compared with 80% of RCTs published in primary care journals.[12] These higher figures for screening eligibility and proportion being eligible in primary care journals need to be viewed cautiously. Higher screening and eligibility may represent under-reporting concerning the processes used to identify the eligible individuals in primary care-based RCTs. Qualitative research has shown that difficulty in recruiting patients is the most frequently mentioned problem in primary care-based RCTs.[13] The administrative processes that are used to identify and quantify the eligible population of patients may be incomplete with the consequence that those RCTs that report complete recruitment of all eligible patients may be based on an under-estimated denominator population. Consequently, the reported median eligibility (65), enrolment (93) and recruitment (54) fractions in this study should be treated with some caution (Table 3).

It has been recognized that patients seen in primary care settings may be significantly different to patients seen in a hospital (secondary or tertiary) care setting. Primary care patients are more likely to have a lower probability of disease; have a milder severity of disease; and have more undifferentiated symptoms.[14] These factors may influence both the estimated benefits and harms from treatment, making the risk:benefit ratio for some interventions less compelling for patients in primary care compared to those in secondary care.[3] More recent evidence has have shown that RCTs published in major medical journals do not always clearly report exclusion criteria.[15] Furthermore, women, children, the elderly and those with common medical conditions are frequently excluded from RCTs; and pharmaceutical-sponsored and multi-centred RCTs in both primary and secondary care are also likely to report higher numbers of exclusion criteria compared to other forms of RCTs.[15] Rather than rely on intuition and judgement, it would be better to include

### Implications

The implications of this study relate to improved reporting and recruitment in primary care-based RCTs, and in assisting planning of future studies. Flow diagrams are associated with improved reporting of RCTs, [16] and implementation of the CONSORT statement has been shown to improve the quality of published RCTs, particularly in relation to dimensions of internal validity.[17] Findings from this study suggest that greater attention to the details of the patient recruitment process may similarly influence and improve reporting of RCTs in terms of external validity.[4,18] There are specific issues that relate to recruitment of patients in primary care that are challenging, such as reliance on family physicians to recruit at the time of a consultation, engaging and running an RCT across many different family practice centres, lack of patient sampling frames and low rates of patient referral.[11,19] More explicit reporting allied to strategies that are known to be important ingredients in terms of improved recruitment- good organization, simplified documentation and study procedures, and anticipating doctor and patient concerns about prior beliefs relating to efficacy and side effects – are issues that need to be considered at the outset when starting up a primary care-based RCT.[20] Complete and accurate reporting of recruitment in published trials will also greatly assist in planning future studies. Researchers can make more realistic estimates of the resources required if the likely eligibility and recruitment fractions are known to be low. Lastly, a low enrolment fraction may not necessarily represent an RCT with poor external validity. If the sample of patients recruited is representative of the eligible population in terms of demographic and clinical characteristics, then external validity is high. This requires investigators to be explicit in describing the recruitment process as well as reporting the characteristics of those eligible individuals included in the RCT and those eligible individuals who were excluded from the RCT.

### Shortcomings

Not withstanding concerns about identification of the full eligible population in some RCTs mentioned above, this study may have other shortcomings. The findings of our study relate to three primary care journals that have a UK/European focus. Only one of the journals selected has a North American focus. It may be that RCTs relevant to primary care are published in general medical and North American based journals and may provide better reporting of issues that relate to external validity. For this reason further assessment concerning the external validity of RCTs in other countries and journals is needed. On a more positive note, investment in primary care research and development in the UK has meant that in absolute terms the number of RCTs published in these three UK journals has nearly doubled compared to a five year period in the early 1990's.[21]

## Conclusion

There is room for improvement in the reporting of dimensions that relate to the external validity of RCTs in primary care, particularly in identification and reporting of the eligible population. Enhancing the external validity of primary care-based RCTs requires improved reporting alongside pragmatic strategies to enhance recruitment and retention.

## Competing interests

RJ and TF were responsible for writing the study protocol. RJ and ROJ extracted data on individual RCTs. CM and AM performed the statistical analysis. All authors were involved with writing and commenting on drafts of the paper.

## Authors' contributions

TF had the original idea for the study and wrote the protocol. RJ and ROJ handsearched and extracted the data. CM and AAM did the analysis. TF wrote the first draft with contributions from all the authors. The authors declare no conflict of interest.

## Pre-publication history

The pre-publication history for this paper can be accessed here:


